# Blocking Rice Shoot Gravitropism by Altering One Amino Acid in LAZY1

**DOI:** 10.3390/ijms23169452

**Published:** 2022-08-21

**Authors:** Shuifu Chen, Yuqun Huang, Jingluan Han, Shijuan Zhang, Qiaoyu Yang, Zhijie Li, Ya Zhang, Runyuan Mao, Ling Fan, Yaoguang Liu, Yuanling Chen, Xianrong Xie

**Affiliations:** 1 State Key Laboratory for Conservation and Utilization of Subtropical Agro-Bioresources, South China Agricultural University, Guangzhou 510642, China; 2 College of Life Sciences, South China Agricultural University, Guangzhou 510642, China; 3 Guangdong Laboratory for Lingnan Modern Agriculture, Guangzhou 510642, China

**Keywords:** *LAZY1*, prostrate growth, shoot gravitropism, subcellular localization, tiller angle

## Abstract

Tiller angle is an important trait that determines plant architecture and yield in cereal crops. Tiller angle is partially controlled during gravistimulation by the dynamic re-allocation of LAZY1 (LA1) protein between the nucleus and plasma membrane, but the underlying mechanism remains unclear. In this study, we identified and characterized a new allele of *LA1* based on analysis of a rice (*Oryza sativa* L.) spreading-tiller mutant *la1^G74V^*, which harbors a non-synonymous mutation in the predicted transmembrane (TM) domain-encoding region of this gene. The mutation causes complete loss of shoot gravitropism, leading to prostrate growth of plants. Our results showed that LA1 localizes not only to the nucleus and plasma membrane but also to the endoplasmic reticulum. Removal of the TM domain in LA1 showed spreading-tiller phenotype of plants similar to *la1^G74V^* but did not affect the plasma membrane localization; thus, making it distinct from its ortholog ZmLA1 in *Zea mays*. Therefore, we propose that the TM domain is indispensable for the biological function of LA1, but this domain does not determine the localization of the protein to the plasma membrane. Our study provides new insights into the *LA1*-mediated regulation of shoot gravitropism.

## 1. Introduction

Plant architectural traits are important targets for crop improvement. For example, in rice (*Oryza sativa* L.), the tiller angle and overall orientation of plants are important factors that influence planting density, light interception, photosynthetic efficiency, disease resistance, and grain yield [1,2]. Rice plants with wide tiller angles have a spreading growth habit yielding favorable access to resources, and they experience less pressure from pathogen attacks. However, this type of prostrate plant occupies more space; thus, leading to a reduction in grain productivity per unit area. In contrast, narrow tiller angles yield compact plants that are inefficient at capturing light and more susceptible to diseases or pests, reducing grain yield [3]. In practice, tiller angle must be balanced between plant performance and grain yield [4,5,6]. Therefore, understanding of the genomic regulatory mechanisms of tiller angle is important for crop genetic improvement that can be achieved through molecular breeding.

A number of genes that determine tiller angle have been characterized in rice, including *LAZY1* (*LA1*) [7,8], *Tiller Angle Control 1* (*TAC1*) [9], *TAC3* and *DWARF* 2 [10], *TAC4* [11], *PROSTRATE GROWTH 1* (*PROG1*) [12,13], *PROG7* [14], *Loose Plant Architecture 1* (*LPA1*) [15], *ONAC106* of the NAC protein family [16], *PLANT ARCHITECTURE AND YIELD 1* (*PAY1*) [17], and *TILLER INCLINED GROWTH 1* (*TIG1*) [18]. Among these genes, *LA1* plays a key role in controlling gravitropic response during the growth of tillers [7,8], and gravitropism is responsible for the orientation of plant organs; thus, contributing greatly to plant architecture [19,20].

*LAZY1*-like genes (designated as *LZYs*) play a highly conserved role in plant gravitropism, but they act on different organs in different plant lineages or at different times during growth, which is likely due to their distinct expression patterns [19,20,21,22]. For example, in rice, *LA1* functions in both coleoptile circumnutation and angling of leaves and tillers [7,8], while its three homologs (namely *OsDRO1*, *OsDRL1*, and *OsDRL2*) primarily affect root architecture [23,24]. In contrast, in *Zea mays*, *ZmLA1* mainly controls shoot gravitropism and inflorescence development [21], and in *Arabidopsis thaliana*, five *AtLZYs* have been identified with distinct functions [22]. In particular, *AtLZY1* is required for gravitropism in the rachis of the inflorescence and in the seedling hypocotyl [25], while *AtLZY2*, *AtLZY3*, and *AtLZY4* redundantly function in root gravitropism, and contribute less to shoot gravitropism [22,26]. Recent studies have shown that although LZYs are uncharacterized proteins, they share five conserved domains, including a predicted transmembrane (TM) domain which was thought to determine their plasma membrane localization [2,7,21]. Despite extensive studies of LZYs, our understanding of their protein structures and the molecular mechanisms governing gravitropism remains limited.

Plant LZYs act as negative regulators of polar auxin transport (PAT), which establishes auxin redistribution after perceiving gravitropic stimulation [7,8,21,22,27,28,29,30,31]. Loss-of-function of *LA1* results in enhanced PAT, and consequently alters the asymmetric distribution of auxin in shoots, leading to a spreading tiller phenotype of rice plants [29]. Expression levels of *LA1* are positively controlled by the gravitropic response factor HEAT STRESS TRANSCRIPTION FACTOR 2D (HSFA2D) [32], which can be directly repressed by two homeobox-leucine zipper transcription factors, OsHOX28 and OsHOX1 [33]. The actions of LZYs and auxin upon gravistimulation have also been confirmed in maize [21], *Arabidopsis* [22], and *Lotus japonicus* [34]. Intriguingly, the dynamic re-allocation of the LA1 protein between the plasma membrane and nucleus is essential for auxin redistribution upon gravistimulation. The interaction of Brevis Radix Like 4 (OsBRXL4) and LA1 at the plasma membrane adjusts the relative proportion of plasma-membrane-localized and nucleus-localized LA1 proteins, leading to asymmetric redistribution of auxin and thus dynamic reorientation of tiller angles [35]. Therefore, elucidating the regulatory mechanisms of LA1 subcellular localization is important for revealing its mode of action upon gravistimulation.

Here, we reported the identification and characterization of a new allele of *LA1* from a spreading-tiller mutant of rice, which was caused by a substitution from glycine to valine occurring in the 74th amino acid position of LA1 that is within its TM domain. The new mutant allele is accordingly named *la1^G74V^*. To elucidate the mechanism underlying the impaired tiller growth in *la1^G74V^*, we first confirmed that the mutation in *LA1* was responsible for the altered architecture via overexpression of *LA1* and *la1^G74V^*. Since the mutation occurred in the TM domain of LA1, we further analyzed the biological function of the TM domain by creating gene-knockout and TM-lacking mutants. We found that these new mutations resulted in altered tiller angle of plants but did not affect the subcellular localization of LA1. Overall, these findings reveal the new insight in the functional domains of LA1 on controlling the tiller angle growth.

## 2. Results

### 2.1. Characterization of the Rice Spreading-Tiller Mutant la1^G74V^

**Figure 1:**
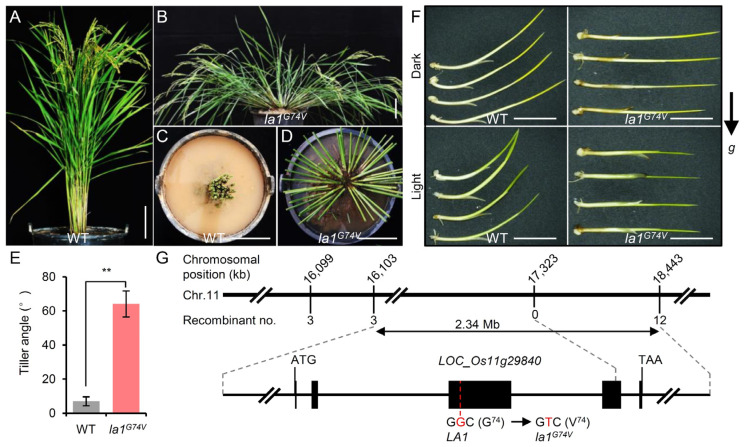
Mapping and phenotype of the spreading-tiller mutant *la1^G74V^*. (**A**–**D**) Plant architectures of the wild-type (WT) plant and the *la1^G74V^* mutant. Scale bars, 10 cm. (**E**) Comparison of tiller angles between WT and *la1^G74V^*. Values are the mean ± SD (**, *p* < 0.01, n = 10). (**F**) Five-day-old seedlings of WT and *la1^G74V^* after 12 h gravistimulation under vertical light (top) or dark (bottom) conditions. The arrow indicates the direction of gravity. g, gravity. Scale bars, 1 cm. (**G**) Fine mapping of *LA1*. *LA1* is located in a 2.34 Mb region between 16,103 and 18,443 molecular markers. Black boxes indicate exons of *LA1*. The marker 17,323 is located in the fourth exon of *LA1*. A non-synonymous G-to-T mutation was identified in the third exon of *LOC_Os11g29840* (LA1), causing a substitution of the 74th amino acid from glycine (G) to valine (V).

To identify key genetic components involved in the control of rice plant architecture, we investigated an EMS-induced mutant library with altered plant morphology [36] and found a mutant that exhibited a spreading-tiller phenotype (Figure 1A–D). Later genetic mapping and functional analysis revealed that this prostrate phenotype was caused by a point mutation located in the *LA1* gene (see below); we therefore named this mutant *la1^G74V^*.

Compared with the wild-type (WT) plants, the *la1^G74V^* plants exhibited an extremely wide tiller angle (Figure 1E). Directional growth of plants is most often associated with either phototropism or gravitropism [37]. Therefore, we first sought to determine whether the wide tiller angle of *la1^G74V^* was caused by impaired phototropism or shoot gravitropism. We planted seedlings of the mutant in a horizontal direction and monitored their response to gravity under light and dark conditions. After 12 h of gravistimulation treatment, the *la1^G74V^* seedlings maintained the horizontal-direction growth under both light and dark conditions (Figure 1F), indicating that their gravitropic response was drastically reduced in *la1^G74V^* mutant seedlings. We further examined the gravitropic response of WT and *la1^G74V^* coleoptiles under dark conditions and found that the coleoptiles of *la1^G74V^* had a reduced gravitropic response compared with the WT (Figure S1). Thus, we believed that the causal gene of the *la1^G74V^* mutant might function in the gravitropic response of coleoptiles and shoots.

### 2.2. Map-Based Cloning of la1^G74V^

To isolate the mutated gene conferring the prostrate growth, we generated an F_2_-segregating population (784 progenies), which was derived from a cross between *la1^G74V^* and *O. sativa* ssp. *indica* ZS97. Among the F_2_ plants, 605 individuals grew normally, and 179 individuals were prostrate, matching a 3:1 Mendelian ratio (*χ*^2^ = 1.96 < *χ*^2^*_df_*_=1_ = 3.84, *p* = 0.05), indicating that the spreading-tiller phenotype of *la1^G74V^* is caused by a single recessive locus.

Based on the genetic linkage analysis with 96 F_2_ prostrate progenies, we preliminarily mapped the candidate locus to a 2.34 Mb region between the molecular markers 16,103 and 18,443 on chromosome 11 and found that no recombination was identified between the 17,323 marker and the *la1^G74V^* locus (Figure 1G). The 17,323 marker was located in the fourth exon of *LOC_Os11g29840* (*LA1*), which encodes a plant-specific protein to control tiller angle upon gravitropism [7,8]. Therefore, we sequenced *LA1* in the mutant and identified a single nucleotide substitution of G-to-T in the third exon of this gene. This substitution occurred at 220 bp downstream of the start codon (ATG) and caused a change in the 74th amino acid, glycine (G), to a valine (V), therefore named this mutant allele *la1^G74V^* (Figure 1G and Figure S2).

**Figure 2:**
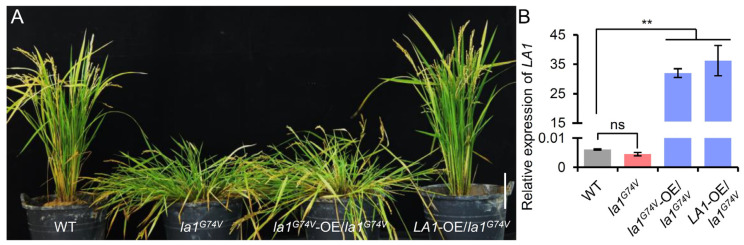
Genetic complementation of *LA1*. (**A**) Plant architectures of WT, *la1^G74V^*, and transgenic *la1^G74V^* plants overexpressing *LA1* or *la1^G74V^*. Scale bar, 10 cm. (**B**) Comparison of *LA1* expression in WT, *la1^G74V^*, and transgenic *la1^G74V^* plants overexpressing *LA1* or *la1^G74V^* via qRT-PCR. *UFC1* served as an internal reference. Values are the mean ± SD (**, *p* < 0.01, n = 3). ns, no significant difference.

To determine whether *la1^G74V^* was the cause of the prostrate phenotype, we constructed the overexpression vectors of the WT *LA1* and mutant *la1^G74V^* driven by the maize *Ubiquitin* promoter, and transformed them into the *la1^G74V^* plants, generating four and three independent transgenic lines, respectively. All transgenic lines (T_1_) carrying the WT *LA1* transgene were rescued and showed a normal tiller angle, while the transgenic lines (T_1_) overexpressing *la1^G74V^* failed to rescue the *la1^G74V^* mutant phenotype (Figure 2A,B). Additionally, we observed no significant difference in the expression level of *LA1* between the WT and *la1^G74V^* (Figure 2B), indicating that the point mutation did not affect the expression of *LA1*. Thus, we concluded that the G-to-T mutation in the *LA1* locus was responsible for the spreading-tiller phenotype of *la1^G74V^*.

### 2.3. The Effect of G74V Mutation on the Conformation and Subcellular Localization of LA1 Protein

**Figure 3:**
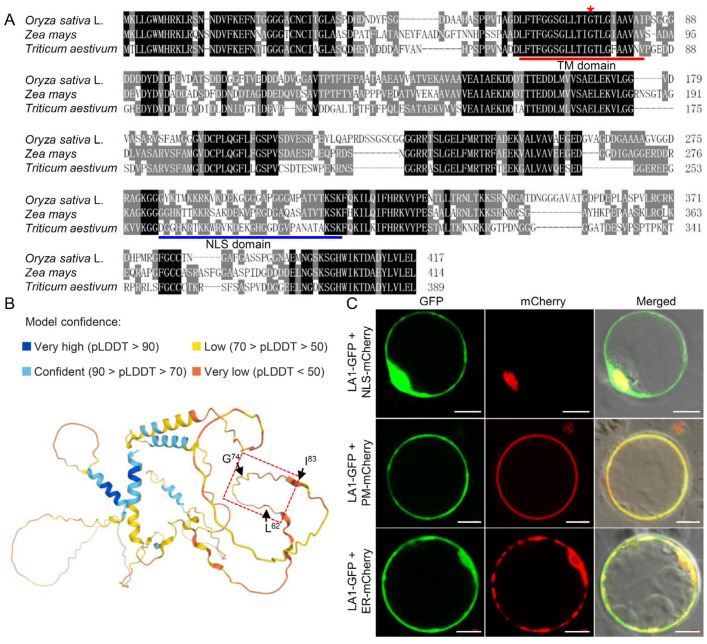
Sequence comparison and subcellular localization of LA1. (**A**) Amino acid sequence alignment of LA1 orthologs from *Oryza sativa* L., *Zea mays*, and *Triticum aestivum*. Underlined sequences indicate the predicted transmembrane (TM) domain (amino acid residues 62–83) and nuclear localization signal (NLS) domain (amino acid residues 281–312). The asterisk indicates the 74th mutated amino position where the substitution mutation has occurred. (**B**) Predicted 3D protein structure of LA1 via the Alphafold Protein Structure Database. The colors represent the per-residue confidence score (pLDDT) between 0 and 100. The dotted box represents the TM domain. (**C**) Subcellular localization of LA1. The LA1-GFP construct was co-expressed respectively with the nucleus-localized NLS-mCherry construct, plasma-membrane-localized PM-mCherry construct, and endoplasmic-reticulum-localized ER-mCherry in rice protoplasts. Scale bars, 10 μm.

Previous studies indicated that the LA1 protein contains a predicted TM domain located from the 62nd to the 83rd amino acid residues, and a predicted nuclear localization signal (NLS) domain (Figure 3A) [35]. Considering that the G74V substitution is located in the TM region, we speculated that this variation might affect the conformation or localization of the LA1 protein. Prediction of 3D protein structure of LA1 showed that the TM domain folded inward, suggesting that this amino acid mutation from the polar glycine to the nonpolar valine might result in a drastic change in the protein conformation (Figure 3B).

We further analyzed subcellular localization of LA1 with the PSORT Prediction program and found that this protein was predicted to localize not only to the nucleus and plasma membrane but also to the endoplasmic reticulum, which plays an important role in gravitropic response [38,39]. To test the subcellular localization of LA1, we performed transient expression analysis using fusion of LA1 to GFP (LA1-GFP) in rice protoplasts and found that the green fluorescence signal of LA1-GFP completely overlapped with the red fluorescence signal markers for the nucleus, plasma membrane, and endoplasmic reticulum (Figure 3C).

**Figure 4:**
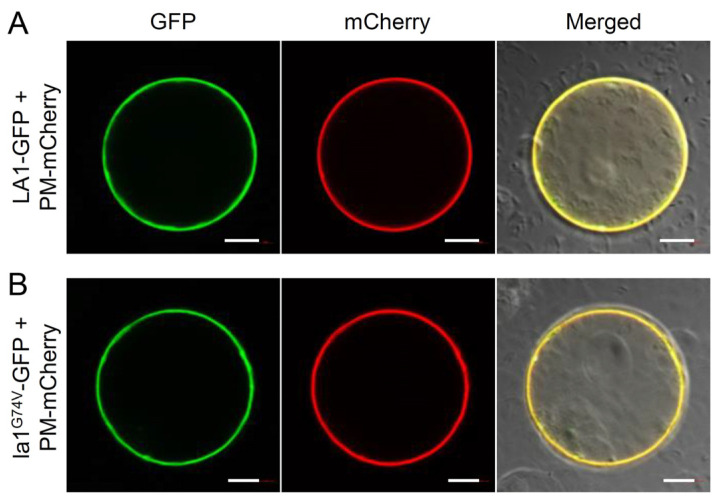
Plasma-membrane localization of LA1 and la1^G74V^. The LA1-GFP (**A**) and la1^G74V^-GFP (**B**) constructs were co-expressed with the plasma-membrane-localized PM-mCherry construct in rice protoplasts, respectively. Scale bars, 10 μm.

Given that the TM domain determines plasma membrane localization of LZYs [7,21,35], we compared the plasma membrane localization of LA1-GFP to that of la1^G74V^-GFP fusion proteins in rice protoplasts. The results showed that both LA1-GFP and la1^G74V^-GFP fusion proteins localized to the plasma membrane with no obvious differences between them (Figure 4A). These results suggest that the G74V variation in the TM domain of LA1 negligibly affects its subcellular localization, but likely eliminates its functional conformation.

### 2.4. The TM Domain of LA1 Is Essential for Its Biological Function Rather Than Its Plasma Membrane Localization

**Figure 5:**
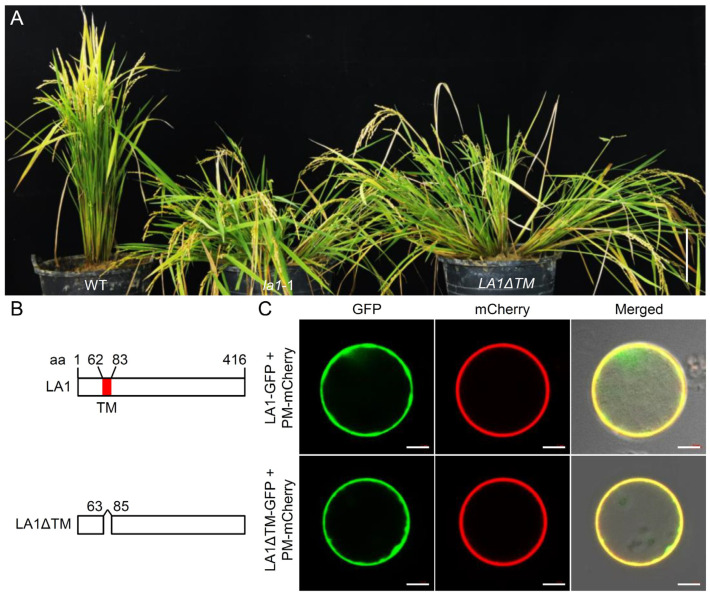
Effect of the TM domain on LA1 function. (**A**) Plant architectures of WT, *LA1*-knockout (*la1*-1), and *LA1-TM-absence* (*LA1ΔTM*) plants. TM, transmembrane. Scale bar, 10 cm. (**B**) Schematic diagram of LA1 and its truncated derivative LA1ΔTM. The red region indicates the predicted TM domain. The folded line indicates the deleted region. aa, amino acid. (**C**) Localization of LA1-GFP and LA1ΔTM-GFP to the plasma membrane. The LA1-GFP and LA1ΔTM-GFP constructs were co-expressed with the plasma-membrane-localized PM-mCherry construct in rice protoplasts, respectively. Scale bars, 10 μm.

To further determine the biological function of the TM domain, we constructed an LA1 knockout (designated as la1) and an LA1-TM-absence (designated as LA1ΔTM) mutant in the Nipponbare cultivar via CRISPR/Cas9-mediated genome editing. To generate the LA1ΔTM mutant, two editing target sites were designed to delete the TM coding region on the third exon of LA1 (Figure S2). Several T_1_ independent lines with homozygous mutations in LA1 were obtained, among which one line carried an LA1ΔTM type allele with a 63 bp deletion that did not cause frame shift but led to the loss of the TM domain (Figure S2; Table S1). Compared to the WT, the LA1ΔTM mutant also showed prostrate growth of plants, mimicking the phenotypes of la1 and la1^G74V^ mutants (Figure 5A). 

We further wondered whether the absence of the TM domain could alter the subcellular localization of LA1ΔTM. Thus, we generated a P_35S_::LA1ΔTM-GFP fusion construct for the transient expression assay in rice protoplasts. Surprisingly, the results showed that removal of the TM domain did not affect the plasma membrane localization of LA1ΔTM-GFP, compared with that of LA1-GFP (Figure 5B,C). These results support the notion that the TM domain of LA1 is essential for its biological function but not for its plasma membrane localization.

## 3. Discussion

LZYs have conserved molecular functions in gravitropic responses, although they share low protein sequence similarity among plant lineages [20,23,40]. For example, AtLZY1 and AtLZY2 share only 19.8% sequence identity, yet *AtLZY2* driven by the promoter of *AtLZY1* can rescue the phenotype of the *atlzy1* mutant [41]. These data support the notion that LZYs may share a conserved functional protein conformation. The functional redundance of LZYs in plant gravitropism is implicated in their five short conserved sequence structures, especially the unique motif “GφL(A/T)IGT” in the TM domain [25]. This is consistent with our findings that a single amino acid mutation is sufficient to eliminate shoot gravitropism (Figure 1). A similar phenomenon was observed in an *AtLZY1^L92A/I94A^* mutant, which contained two amino acid mutations within the TM domain [42]. In addition, prediction of 3D protein structures revealed that the TM domain may be vital for the functional conformation of LA1 (Figure 3B). In view of that LZYs are novel proteins that show no homology to any other functionally characterized proteins [7,20,23,40]; future studies will be pivotal to clarify their nature characteristics and modes of action.

Subcellular localization of a protein is essential for understanding its molecular mode of action. Emerging studies have demonstrated that subcellular localizations of LZYs are divergent. For example, while LA1, ZmLA1, and AtLZY1 localize to both the plasma membrane and nucleus, OsDRO1, OsDRL1, AtLZY2, AtLZY3, and AtLZY4 primarily localize only to the plasma membrane [21,23,26,34,41,42]. Recent studies showed that the NLS domain determines localization of LA1 to the nucleus [35,43], while removal of the first 100 N-terminal (containing the TM domain) or last 96 C-terminal amino acids (not containing the TM domain) eliminates its localization to the plasma membrane [7,35]. Nevertheless, Zhu et al. [43] showed that the absence of 160 C-terminal amino acids of LA1 does not impair its localization to the plasma membrane localization, while in maize, the TM domain was found to determine ZmLA1 localization to the plasma membrane [21]. Despite the high amino acid similarity of TM domains among LZYs (Figure 3A), the evidence that deleting the TM domain of LA1 did not impair its localization to the plasma membrane (Figure 5B,C) supports the possibility that functions of the TM domain within this protein family may be highly divergent. Similarly, OsBRXL4 regulates shoot gravitropism by affecting PAT as did LA1, and its interaction with LA1 at the plasma membrane enhances the relative proportion of LA1 protein localized to the nucleus [35]. Subcellular localization of LZYs seems to be dynamic and regulated by their signal peptides as well as interactors; for example, Dong et al. [21] found that ZmLA1 in maize could interact with the auxin transport regulator protein kinase, ZmPKC, at the plasma membrane and the AUX/IAA-related protein, ZmIAA17, in the nucleus. Intriguingly, the TM domain of ZmLA1 is associated with its interaction with ZmPKC at the plasma membrane, but seemingly does not affect its interactors in the nucleus [21], suggesting that the TM domain may specifically participate in the localization of its auxin-related interactors to the plasma membrane. Taken together, these observations regarding an association between LZYs and proteins involved in auxin redistribution at the plasma membrane compared with the nucleus invite further investigation.

The endoplasmic reticulum serves as an important Ca^2+^ storage organelle and is thought to regulate gravity perception and signal transduction through triggering release of Ca^2+^ [38,39,44,45,46,47]. A recent study found that the regulatory pathway “protein processing in endoplasmic reticulum” is activated early in the gravitropic response, and that the Ca^2+^ response factor HSFA2D acts as an upstream positive regulator of the gravitropic response mediated by LA1 [32]. Consistent with these findings, our results showed that LA1 could localize to the endoplasmic reticulum (Figure 3C). Thus, it appears that LA1 may play an important role in Ca^2+^-mediated signal transduction upon gravistimulation, and this merits further study.

Natural genetic variations in many genes affect the response of plants to various environmental conditions by altering their expression or producing protein variants [48]. For example, genetic variations in some rice genes contribute greatly to the change in growth habit from prostrate in wild rice to the erect in cultivated rice, which consequently produces higher grain yields [9,12,13,14,49]. The differences in the tiller angles between *indica* and *japonica* cultivars are due to the four-nucleotide substitutions in the 3′-untranslated region of *TAC1*, which reduces *TAC1* expression in *japonica* and reduces tiller angle [9]. Previous studies have indicated that *LA1* barely affects other agronomic traits, making it a desirable target for improving yield without unwanted consequences [7,8]. For genes such as *LA1*, precise genomic editing in crops can accelerate crop improvement in a targeted manner [40,50,51]. Therefore, it is feasible and worthwhile to obtain favorable alleles of *LA1* for breeding improvement through editing of vital *cis*-elements or amino acids.

## 4. Materials and Methods

### 4.1. Plant Materials and Growth Conditions

The *la1^G74V^* mutant was obtained from an ethyl methane sulfonate (EMS)-induced *O. sativa* ssp. *japonica* Nipponbare mutant library [36]. We generated an F_2_-segregating population derived from a cross between *la1^G74V^* and an *O. sativa* ssp. *indica* Zhenshan 97B (ZS97) for fine mapping. The plants were planted in paddies under natural growing conditions in Guangzhou. The primers used for map-based cloning are listed in Table S2. 

### 4.2. Analysis of Plant Gravity Response

We performed a gravity response assay described by Li et al. [7]. In brief, the gravity response of rice was investigated using five-day-old seedlings planted in plates with 1/2 MS medium (pH 5.8) under light and at 28 °C, and the plates were placed in an orientation to keep the seedlings vertical to the direction of gravity for at least 12 h. 

### 4.3. Sequence Alignment and 3D Structural Modeling of Proteins

The full-length amino acid sequences of LZY family proteins were aligned with BioXM2.7.120 (https://cbi.njau.edu.cn/BioXM/, accessed on 20 January 2022). Modeling of three-dimensional (3D) protein structure was performed via the Alphafold Protein Structure Database (https://www.alphafold.ebi.ac.uk/, accessed on 12 March 2022).

### 4.4. RNA Extraction and qRT-PCR

Total RNA was extracted from rice seedling leaves with TRIzol reagent (Thermo Fisher Scientific, Waltham, MA, USA), and reverse-transcribed into cDNA using the TranScript One-Step gDNA Removal and cDNA Synthesis SuperMix (Transgen, Beijing, China). qRT-PCR was performed using a ChamQ Universal SYBR qPCR Master Mix kit (Vazyme, Nanjing, China) and the CFX Connect Real-time PCR Detection System (Bio-rad, Hercules, CA, USA) with three biological replicates. For downstream analyses of qRT-PCR results, *UFC1* (*LOC_Os10g13800*) was used as an internal reference based on findings by Zhao et al. [52]. Relative expression was calculated using the 2^−Δ*C*t^ method [53]. The primers used for qRT-PCR are listed in Table S2.

### 4.5. Vector Construction and Rice Transformation

To generate *P_Ubi_::LA1* and *P_Ubi_::la1^G74V^* constructs for the genetic complementary analysis, the coding sequences of *LA1* and *la1^G74V^* were amplified using the primer pair *LA1*-OE-F/*LA1*-OE-R (Table S1) and cloned into an overexpression vector. Recombined vectors driven by the maize *Ubiquitin* promoter were introduced into *Agrobacterium* and then transformed into the *la1^G74V^* mutants. The design of primers for the target sequences, and a vector for CRISPR/Cas9-mediated genomic editing of LA1, and the decoding of targeted mutation events, were performed with CRISPR-GE (http://skl.scau.edu.cn/, accessed on 16 May 2021) [54]. For deleting the TM-coding region of *LA1* or knocking out *LA1*, we constructed a CRISPR/Cas9 editing vector as described by Ma et al. [55]. We introduced the recombined vector into *Agrobacterium* and then transformed into Nipponbare. The primers used to generate the transformation vectors are listed in Table S2.

### 4.6. Subcellular Localization

Subcellular localization prediction of a protein was performed with PSORT Prediction (http://psort1.hgc.jp/form.html). The coding sequences of *LA1*, *la1^G74V^*, and *LA1ΔTM* were amplified using the primer pair *LA1-GFP*-F/*LA1-GFP*-R (Table S2) and cloned into the N-terminus of the green fluorescent protein (*GFP*) gene in the pLYd1GFP vector. The recombinant vectors, which were driven by the *35S* promoter, were co-expressed respectively with the nucleus-localized construct, *P_35S_::NLS-mCherry*, the plasma-membrane-localized construct, *P_35S_::PM-mCherry* or the endoplasmic-reticulum-localized construct, *P_35S_::ER-mCherry* in protoplasts of rice leaf sheath. This procedure was described in greater detail in Han et al. [56]. Following the transformation with *GFP*, we visualized and imaged the fluorescent signals of the transformed cells using a confocal microscope (Zeiss, Oberkochen, Germany).

## 5. Conclusions

In the present work, we identified and characterized a novel allele of *LA1*, *la1^G74V^*, which harbors one non-synonymous mutation in the TM-domain coding region, and we investigated the biological role of the TM domain using this allele. Our results showed that mutation or removal of the TM domain resulted in loss of shoot gravitropism, but negligibly affected the localization of LA1 to the plasma membrane [7,21]. We also found that LA1 could localize to the endoplasmic reticulum, an important organelle that controls gravitropic response of plants via triggering Ca^2+^ release. Our findings provide new insights into subcellular localization of LA1 and suggest that the dynamic allocation of LA1 between organelles may contribute greatly to plant gravitropism control.

## Supplementary Materials

The following supporting information can be downloaded at: https://www.mdpi.com/article/10.3390/ijms23169452/s1.
