# Supplementary material for: Blocking Rice Shoot Gravitropism by Altering One Amino Acid in LAZY1
Source: Int J Mol Sci. 2022 Aug 21;23(16):9452. doi: 10.3390/ijms23169452 (PMC9409014; doi:10.3390/ijms23169452)
Supplement: Supplementary file 1 [file ijms-23-09452-s001.zip › ijms-1826464-supplementary.pdf]

Supporting information

Blocking rice shoot gravitropism by altering one amino acid in LAZY1

Shuifu Chen [1, 2, †], Yuqun Huang [1, 2, †], Jingluan Han [1, 2], Shijuan Zhang [1, 2], Qiaoyu Yang [1, 2], Zhijie Li [2], Ya Zhang [2], Runyuan Mao [2], Ling Fan [2], Yao-Guang Liu [1, 2, 3], Yuanling Chen [1, 2, *], Xianrong Xie [1, 2, 3, *]

The following supporting information is available for this article:

Figure S1. Gravity response of the wild-type and *la1*^{G74V} coleoptiles.

Figure S2. Genomic sequence of *LA1* and its upstream region.

Table S1. Genotypes of the CRISPR/Cas9-editing mutants of *LA1* (T_1).

Table S2. Primers used in this study.

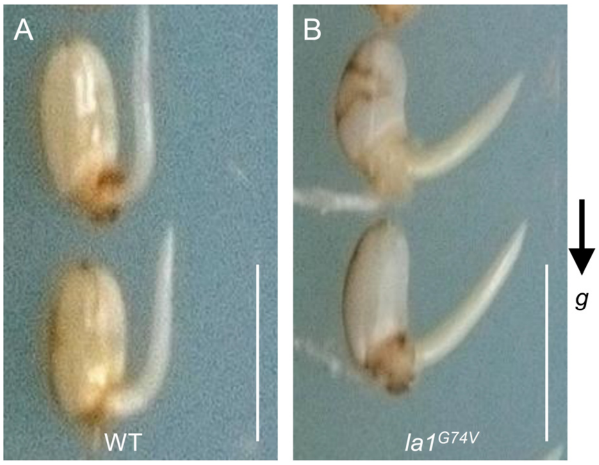

Figure S1. Gravity response of the wild-type and *la1*^{G74V} coleoptiles.

(**A**,**B**) One-cm-length coleoptiles of wild-type (WT) and *la1*^{G74V} rice after six-hour gravistimulation under dark conditions. The arrow indicates the direction of gravity. *g*, gravity. Scale bars, 1 cm.

ATCTTAGCACGCTAAACCGGCTTCCAAG**ATG**AAGGTTAGTGTTTCATCTATTGAACATATCCATGCTTA
TTAGTTTCGTAATGTAGATACTAAGTTTATGTATATGTAGTTCTCTTTTGGAACTTGAGTTGTTAATTT
CTTTTTGTAAATATTTTTTGTTTGGTTTGGTTACGTTGCTGGCCTAGCTGATCATGCTTTCTGGGTTGA
ATAAATGTAGCTACAACTATTAATCCTTTATTTTGTTTTTCCATCATTGCCGTTGTCATCATCTTTCAT
TGTCATCATCATCTTCTCCTTTTGACGATCTTCATCAGATCTTGATCAAAAGTAATAGACCATGCATTT
TCATGATTGATGAAGTTCATAAAAATTTCTGATCCTCATATGTGTATATGATCAGCTCTTAGGTTGGAT
GCATCGAAAGCTACGGAGTAATAATGACGTGTTCAAAGAGTTCAACACCGGAGGAGGTATGCACGCACA
ACTTAATTAATCTATTTTTTGTCCTTTTTTTCTTATGTTTAATTGATGAAGTATAATTGTTTTGAAAAA
TACAGTAGCTCGTAACACAAACGCACTCACCTCTAGTACTCTCTCCAGTGTTTTTATATATTAAACTAA
CGTCATATAAAAAATTGAAGTTAGTATATATGTATAATAGTTCATCGGTACATGCAGAAAAAAAAAAGA
GTTTGTCTCTGGTTTTAGATCTGTAAGCATGCCCACAATAATGAGAAAACCACTGCAAAATTGTTGGCC
GGCCGGTCCCACATGTCATTGACCAGATTAGCCGTCTGAAGTGCTGAACTGTATGGAACACTAGCAATC
ATCAATGGGAGTTTTGCACTCTTGCGCTTGGTCACATTGGAGATGGCACATCTATCTATAATATCACAC
ATGCCACTTTTCTTCCAGCCTCAATATCTATAGTTCCAGCACTAGCTAGCTAGCTAGACCAACTATTGC
ATAGCAAGAACCCTATATTATCATTTTGCAGATAACATTTTCTTTTCCATGTAGTAACATTGCCATGGA
AAGATCACGAGGGCATGTCTATCTTTTGTGAAACTCTCTCTCTCTCCTTTTGAAGGCTTCCTTTGAGAG
TTTTCTCCTTGTTGGACTGTTGCTCTTAGGCTCAGCATGTTAATATCAGAGTTTTCGCCCGTTTTTCAT
AAATCAATTGTGCCGTTGTAAGATTTTTGCGTCCAGTTTTCCTTATAAACTGGACCTCTCTGCCTGTTT
CCTCGAGAAAAATTGTCCAAGAGCATTCTCGACGATTCAACAACTAGAATTCAACAAATAAAGATTTTT
ATATATGGTGCATATGGTACGGTGAGATATTGTATTTTTAGTGTGAGATACCAAAAGAGCTACAACAAA
TTAATACGATAAAATAAGGGAAATTTCATGGACTAACTAGTTAGAAACATATTTTGAGTAAATGAAAAT
TTTATTTTATTACCTTGGTTTACAAAAgacttataataagtgctgtcaaacatctaaaatgttaaattc
ttaatagacgaaaagtattagaatttaaaacgtgaaaattatatactttttgatgaaaaatagtaggaa
tgtgaaaattatattagtagactttttttgatgaaaactctttcatatagttatatgttaatttttat
aactatatagtttgagaaagtaatcatataacttttgcattaaaaaatgtgtcaatgtccaaaacATTA
TCTTAATTAACCCGGTCCTTCTCCTCCCAAAGGAACACATAAAAAAGGACAAAAACCAATACATCTTGA
AAAACTTACATAGAAAAAGCTTAATCATATTTTATATCACCCTGATCCCGTTGCAACGCACATGAATGT
AACTATTTCTTTTTAAAATGGTGTTGTACTTCATAAATTTGAAACAAAAAAAATACATATAATGTAACC
CTTTTAATTTCTGAAACACATCAAAACTTTTCTTTCTCAAAAAAAAGAAAAGAAAACACATGAAAGCCT
GCATAATTTTGCATGCAAATGATCGtactccttccttttaaggttacaagactttcttacattaccca
aatttatatagataataaatctagacacaaatatatgtgatttattaatatgtatatgaatgtgaacaa
tgccaaaaagtcttataatataaaatggagaaagtaTTTaatactccctcagtttctaaatatttgaca
ccattgatttttaaacatgtttaatcattcgtcttattcaaaaatttaagtaattattaattatttt
cctatcatttgattcattgttaaatatacttttatgtatacatataattttacgtatttcacaaaagtt
tttgataagacggacggtcaaacatgtgctaaaaagtcaactgtgtcagatatttagaaacggagggag
taATTCACTGTGTGACTGCAGGTGGGGCCTGCAACTGCATCACCGGGCTTGCCTCGCCTGACCACGACA
ACGACTACTTCTCCGGCGACGACGCCGCCCACGCCTCGCCGCCGGTCACCGCCGGCGAC**CTCTTCACCT**
TCGGCGGCAGCGGCCTTCTCACCATCGGCACGCTAGGCATCGCCGCCGTCGCCATTCCCAGCGGCGGCG
ACGACGACGACTACGACATCGACTTCGAGGTGGACGCCACCAGCGACGACGACGGCGGCTTCACCGTCG
AGGACGACGACGCCGACGTCGGCGGCGCCGTCACGCCCACCTTCACCTTCCCCGCGGCGACGGCGGCGG
AGGCGGTCGTCGCCACCGTGGAGAAGGCAGTGGCCGCGGTGGAGGCGATCGCGGAGAAGGACGACGACA
CCACCACGGAGGACGACCTGATGGTGGTGAGCGCCGAGCTGGAGAAGGTGCTCGGCGGCGTCGACGTGG
CGTCGGCGCGGGTGAGCTTCGCCATGGGCGGTGGCGTCGACTGCCCGCTCCAGGGCTTCCTGTTCGGCT
CCCCGGTGAGCGACGTCGAGTCGCGCCCGGAGTACCTGCAGGCGCCGCGGGACTCGTCCGGCTCCTGCG

GCGGCGGCGGGCGGCGCACCTCGCTCGGCGAGCTGTTCATGCGCACCCGCTTCGCCGACGAGAAGGTGG
CGCTCGTCGCCGTCGCCGAGGGCGAGGACGGCGTCGCCGGCGACGACGGCGCTGCTGCTGCCGGCGTCG
GCGGAGACAGAGCGGGGAAAGGCGGCGGCTACAAGACGATGAAGAAGAGGAAGGTGAAGGACGAGAAAG
GCGGCGGCGGCGCCGCCGGCGGTGGAATGCCGGCGACGGTGACGAAGAGCAAGTTTCAGAAGGTAACTT
TTTTTTTTGTTGTTGCTAGCATTTTAATTTGCTTTGAAGAAAACTATAAAATATCTGCAATTTTGGCTG
ACTATTTCATGAGGTTTCCTTGGATATGATCATTTGATTAATTAGTTCGTTCGATTGCTATAGTTTTAG
TTCATTTTTCTTTAATTAATTTGTAATAAAACTCAAGAGTTATTGTGAGTTTTTTTTTTATTATTTTTG
CTTCAGTACGTGCATTTTCTACTAGTGAAACTTCAATATTAACCAGGCCAGTCGACATTTCTTAATTGT
GATTTGATTTAAGGGTCAAGATTAGAACTATAGATTGAATATGCAAAGTTTCAGCTCGGACGACATTTG
AACATTTGACTAATTATATATTTCCGAGTTCAATTGTTTGCCGGTAAAATTCTGAACTTGCAGTTGGAA
CATTCAAGATTCAACTCTGATTCACATGATCTCGAACTCAATATCTCCAACTTTATAAAAGACAAAAA
GATTACTGCTCAACGGTGAGTTTAATGAAGGAATGCTCATCACAGCAAATCCATAATTCTATATAACTC
TCTGTAATATGTAATGGTAAGAAACAGATATTCATGATTAAATCATTTTTCCCCTTCAGAAAGAAATAC
TGATTTTTCGTGGTACATCCAAAATTACTCCAAAGTTTACACTGAATTTCACGCTGTTCTTGGTGTTTA
AGATTAATTTTAGGTAGATATTTTCTAGTCCCGCAACAAAGCTGACAATGAAAACCCCAAAACTAATCC
TAAATGAAATTCTAAAATTAAGAAAAACAGCTTTAGATTATAAATTTTAATAGTAAGTCCAAGGAAATA
GCCAGCAATGTTAATAAGATTGCTGATAAGAATATATAACAGTTTGGAGCCAATCCATTTCACATGTGT
ATCCTGCTCTGGGACATAAGTAGACACAAATCCCGAGTTTTGACTATCATCTCATAATCGGAAGATCTT
TCACTGTGTCGTCAGATTCGAGTTAAGTTTCTGTAGACCAACTTGGTGTGTCAATGGTGTTGTTAGGAG
TGGGGATACTGGGGGCAGTGAGTTGACATGCGATTTGTTTGCAATCTCCTGTCTAATGCTATCTTTATG
GATCATTTCATTTGTCATTTTTCGTTTGCCCCATAAACAACAAGGGATTCTTGATTTGATATATTATAC
ATCCTTCTAGGTGCTCTCTTGCATTTCTTTTCCTTGGAGAGATGCTCCAAATATCCCTCAAAAGAAATT
CTAGATTCCTTTTGCTACTACTTGGTACTTACATTTAATTTTGTTACAACGAATGAAAGAAACTGAGTT
TCAGAAAGTGATCAAAAGTTGACAATGTTCTGAAATGAGGAAACATCTGCTCATTGCAGATCCTTCAAA
TCTTCCACAGGAAAGTCTACCCCGAGAACACACTCCTCACAAGGAATCTGACCAAGAAGAGCCGCAACC
GCGGCGCCACCGATAATGGCGGTGGCGCCGTGGCCACCGGAGACCCCGACGGGCCTCTGGCCTCGCCGG
TGCTCCGGTGCCGGAAGGACCATCCCATGAGGGGCTTCGGCTGCTGCACCAATGGCGCCTTCGGTGCAT
CGTCACCGGGAGGCAACGCCGAGATGAACGGCAACAAGAGCGGCCACTGGATCAAGACTGATGCCGACT
GTGAGTAGCACTGCACACCTTGGTGTCTCCATCCATCTTCAATGGATCTATCTTTGCAATCATGCATTC
AGTCTTGACAGCTACATCTTTCCATACTATTTTTGGGGAATCTTCCAAGAGCTATCCATCACTAGTTTC
TGAGTTACTGTGTGCTGATGCACACACTGCAAACATTGTGCTTTTTCACTGAAACCCTGTCTCTCTGAT
CTGATGCAGACTTGGTGCTGGAATTA**TAA**TGGGGAAAAGAAGAGGAGCTATTTGTTTCATCAAGAATTA
AAGCTTGAATAGTGAGCATCTCATATATATGAATATGTGCTTGCTGTTACAATATAATCTCTATCTGTT
TGGTATAGAAGGGCTTGAATGTGCTGTATATGTCACTATATATTGGGGGGGAGAGGATTACTATGAGAT
CAACTCATAAGTGTGTGGTGTTTATATACATTGCTCTGTGAATGTATGACAGAGATCAGAACTTAATGT
ATTGTATGCTTTCTTGATGTGATTGGTGTTTATGAGCCTAGCTGATGGCAATATTGTGTGTGGGTCCAC
CCCT

Figure S2. Genomic sequence of *LA1* and its upstream region.

The exon sequences are highlighted in yellow. The UTR sequences are shaded in gray. Bolded letters indicate the predicted transmembrane domain coding region. The red letter indicates the non-synonymous G-to-T mutation. Boxed letters indicate the two protospacer adjacent motifs. Underlined letters indicate the two CRISPR/Cas9-editing targets.

Table S1. Genotypes of the CRISPR/Cas9-editing mutants of *LA1* (T_1).

T_1 plant		Target mutation (3′– 5′)	Mutation type
la1-1	Ref	CGCTGCCGCCGAAGGTGAAGAGG	No mutation
	Allele1	CGCTGCCGCCGAAGGTGAAGAGG	
	Allele2	CGCTGCCGCCGAAGGTGAAGAGG	
		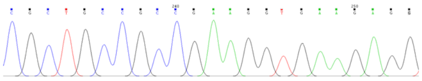	
	Ref	CCGCTGGGAATGGCGACGGCGGC	Homozygous
	Allele1	CCGCTG-GAATGGCGACGGCGGC	
	Allele2	CCGCTG-GAATGGCGACGGCGGC	
		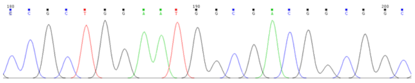	
la1-2 (*LA1ΔTM*)	Ref	CCGCTGG--//--GGTGAAGAGG	Homozygous
	Allele1	CCGCTG---63 bp---AAGAGG	
	Allele2	CCGCTG---63 bp---AAGAGG	
		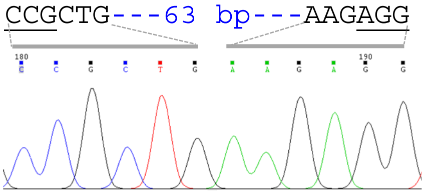	

Note: Ref is the wild-type reference sequence. Underlined bases indicate the protospacer adjacent motifs. Short blue lines indicate deletion of base-pairs. bp, base-pair.

Table S2. Primers used in this study.

Primer Name	Sequence (5′– 3′)
Molecular markers for the map-based cloning	
16099-F	GTCTAGTACTATTAGTACTAG
16099-R	TTTGTACACATCATATACAAAG
16103-F	TAGGCGATAAAATGAAAGAC
16103-R	ATAGAAAACTACACAAGTAC
17323-F	TACCCCGAGAACACACTCCT
17323-R	AGGGGCTTCGGCTGCTGCAC
18443-F	TGTTTTGCTGCAGCTTTTCT
18443-R	CTCTACGCAGAAGCAAGGCT
Construction of the overexpression vector	
LA1-OE-F	ACTTCTGCAGGGTACATGAAGCTCTTAGGT
LA1-OE-R	AATTCACACTTGTAGTTATAATTCCAGCAC
Construction of the CRISPR/Cas9 editing vector	
LA1-TM-gRT1	CGCTGCCGCCGAAGGTGAAGGTTTTAGAGCTAGAAAT
LA1-TM-OsU6aT1	CTTCACCTTCGGCGGCAGCGCGGCAGCCAAGCCAGCA
LA1-TM-gRT2	CCGCCGTCGCCATTCCCAGGTTTTAGAGCTAGAAAT
LA1-TM-OsU6bT2	CTGGGAATGGCGACGGCGGCAACACAAGCGGCAGC
Construction of the subcellular localization vector	
LA1-GFP-F	TTACAACCTCACTCAAGTCCGTTAGAGCCCATGAAGCTCTTAGG TTGGATGCATC
LA1-GFP-R	GAGTATTCTTAGGTGGCAGCGAACGAGCCCTAATTCCAGCACCA AGTAGTCGGCA
qRT-PCR	
UFC1-qRT-F	GATGGCAAGACCCACAAG
UFC1-qRT-R	TCCCGAACCTTGGGCAGT
LA1-qRT-F	GAGCAAGTTTCAGAAGATCC
LA1-qRT-R	TCCTTCCGGCACCGGAGCAC
